# Clarifications of the Motor Level Definition in the International Standards for Neurological Classification of Spinal Cord Injury in Not Clinically Testable Myotomes

**DOI:** 10.46292/sci24-00094

**Published:** 2025-08-22

**Authors:** Christian Schuld, Steffen Franz, Laura Heutehaus, EMSCI Study Group, Kristen Walden, Gianna Rodriguez, James Guest, Fin Biering-Sørensen, Steven Kirshblum, Ruediger Rupp, European Multicenter Study about Spinal Cord Injury (EMSCI) Study Group, Rainer Abel, Armin Curt, Axel Hempfing, Yorck-Bernhard Kalke, Jiri Kriz, Doris Maier, Martin Pouw, Norbert Weidner

**Affiliations:** 1 Spinal Cord Injury Center, Heidelberg University Hospital, Heidelberg, Germany; 2 Medical Faculty Heidelberg, Heidelberg University, Heidelberg, Germany; 3 AUVA Rehabilitation Center Weißer Hof, Department for Spinal Cord Injury, Austria; 4 Praxis Spinal Cord Institute, Vancouver, Canada; 5 Department of Physical Medicine and Rehabilitation, Michigan Medicine, Ann Arbor, Michigan; 6 The Miami Project to Cure Paralysis, Miller School of Medicine, Miami, Florida; 7 Department of Spinal Cord Injuries, Rigshospitalet, University of Copenhagen, Denmark; 8 Kessler Institute for Rehabilitation, Department of Physical Medicine and Rehabilitation, West Orange, New Jersey; 9 Rutgers New Jersey Medical School, Newark, New Jersey; Spinal Cord Injury Center, Klinik Hohe Warte, Bayreuth, German; Spinal Cord Injury Center, Balgrist University Hospital, Zurich, Switzerland; Center for Spinal Cord Injuries, Werner Wicker Hospital, Bad Wildungen, Germany; RKU Universitäts-und Rehabilitationskliniken Ulm, Ulm, Germany; Spinal Cord Unit, Department of Rehabilitation and Sports Medicine, 2nd Faculty of Medicine, Charles University and University Hospital Motol, Prague, Czech Republic; BG Unfallklinik Murnau, Murnau, Germany; Department of Orthopedic Surgery, Radboud University Medical Center, Nijmegen, The Netherlands and Department of Orthopedic Surgery, Sint Maartenskliniek, Nijmegen, The Netherlands; Heidelberg University Hospital, Spinal Cord Injury Center, Heidelberg, Germany

**Keywords:** EMSCI, ISNCSCI, motor follow sensory, motor levels, simulation

## Abstract

**Background:**

In the International Standards for Neurological Classification of Spinal Cord Injury (ISNCSCI), two approaches for determining motor levels (MLs) in not clinically testable myotomes (C2-C4, T2-L1, S2-S5) are described: one where the motor level follows the sensory level (MFSL) and another deriving motor function from sensory function (MFSF). Their results differ when (1) all key muscles of an upper (or upper and lower) extremity are scored as intact, (2) sensation is not normal in key muscle segments, and (3) a contiguous region of normal sensation starts at T2 (or S2).

**Objectives:**

This work aims to characterize these cases and to discuss explanations.

**Methods:**

We analyzed 1330 early and late ISNCSCI assessments of 665 individuals from EMSCI.

**Results:**

Forty-nine (3.6% of all 2660 MLs) MFSL (63.3% T1, 36.7% S1) and MFSF MLs from 34 individuals differed without consequences on ASIA Impairment Scale (AIS) grades (4 AIS A, 1 AIS B, 29 AIS D). In 16 AIS D cases, all testable motor functions were intact, with a mean Spinal Cord Independence Measure (SCIM) total score of 95.67 ± 3.51 in 3 individuals with MFSL-ML T1 and 100 in 5 individuals with MFSL-ML S1. The MFSF-MLs are on average 9.63 ± 7.50 (T1: 12.16 ± 8.43; S1: 5.28 ± 1.36) segments caudal to the sensory level (SL).

**Conclusion:**

We identified and characterized rare cases with an unusual sensory impairment pattern, which could be explained by an isolated damage of afferent spinal tracts or the presence of non-SCI conditions. Further investigations of these case are necessary for a more conclusive ML definition.

## Introduction

Standardized classification of spinal cord injury (SCI) according to the International Standards for Neurological Classification of Spinal Cord Injury (ISNCSCI)^1^ is essential for clinical decision making, for assessing the course of changes in sensorimotor functions, and in researching characterization and stratification of study participants and definition of endpoints. Most importantly, as an inclusive bedside assessment, ISNCSCI enables communication among clinicians worldwide. ISNCSCI includes a comprehensive set of classification rules with precise instructions on how to correctly determine the sensory and motor levels, the American Spinal Injury Association (ASIA) Impairment Scale (AIS) grade, and the zones of partial preservation. Although examiners substantially improve their classification competency with training,^2^ the correct determination of motor levels represents the most difficult classification step.^3^-^5^ This difficulty arises because muscle functions can only be assessed by a manual testing of 10 key upper and lower extremity muscle functions corresponding to the myotomes C5-T1 and L2-S1. For all other spinal segments (i.e., C2-C4, T2-L1 and S2-S5), no muscle function is tested as part of the ISNCSCI motor examination. In clinically testable myotomes, the motor level (ML) “is defined by the lowest key muscle function that has a grade of at least 3, providing the key muscle functions represented by segments above that level are judged to be intact (graded as a 5)”^1^ (called the *key muscle* rule in this article). However, “for those myotomes that are not clinically testable by a manual muscle exam, i.e., C1 to C4, T2 to L1, and S2 to S5, the motor level is presumed to be the same as the sensory level if testable motor function above (rostral to) that level is normal as well.”^1^,^6^ This ISNCSCI classification rule is colloquially referred to as the *motor (level) follows sensory level* (MFSL) rule. The analysis of classification tests conducted after ISNCSCI training courses shows that approximately 70% of all ML misclassifications relate to the cases in which the MFSL rule applies.^3^,^7^

For further clarification of the MFSL rule, another approach was introduced in 2003, where motor function in not clinically testable segments is derived from sensory function of the same segment, that is, “If the sensation for a segment is normal, motor function for that segment is considered normal; if sensation is impaired, motor function is considered impaired.”^8^ In this work, we call this the segmental *motor follows sensory function* (MFSF) approach. A specific scenario for this rule is the motor level determination in the *transition zones* C4/C5 and L1/L2, which was introduced in the 2009 review of ISNCSCI.^9^ Although the MFSF approach is not explicitly stated in the most recent ISNCSCI revision, it is implicitly part of examples 2 and 3 in the motor level section of the most recent edition^1^: “If the sensory level is C4, with the C5 key muscle function strength graded as ≥3, the motor level would be C5 because the strength of C5 is at least 3 with the ‘muscle function’ above considered normal; presumably if there was a C4 key muscle function it would be graded as normal since the sensation at C4 is intact” and “If the sensory level is C3, with the C5 key muscle function strength graded as ≥ 3, the motor level is C3. This is because the motor level presumably at C4 is not considered normal (since the C4 dermatome is not normal), and the rule of all levels rostral needing to be intact is not met.”^1^

In most cases, where the ML is expected to be in a segment with not clinically testable motor function, both the MFSL approach plus application of the 2009 transition zones approach described above and the MFSF approach result in the same ML. However, there are constellations with regions of normal sensory and motor function below the neurological level of injury (NLI) where no clear guidance on ML determination is given in the ISNCSCI.^1^ As a consequence, the ASIA International Standards Committee—responsible for updating and revising the ISNCSCI—is recurrently approached to provide support in correct classification of such cases.

**Figure 1.**
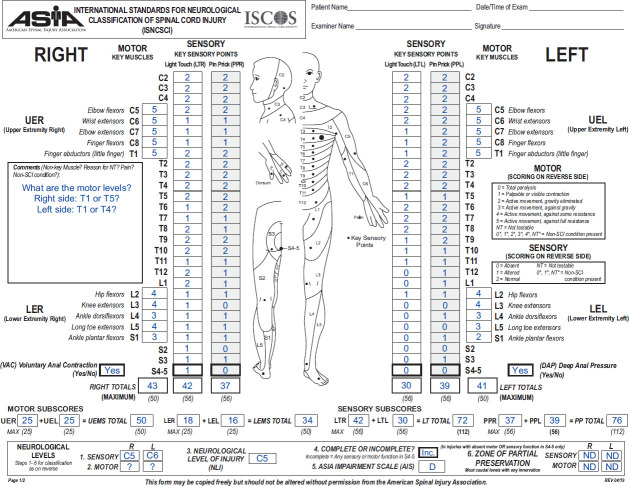
An International Standards for Neurological Classification of Spinal Cord Injury (ISNCSCI) sample case with uncertainties regarding the determination of the motor levels. The Praxis ISNCSCI algorithm calculator,^11^ which implements the motor follows sensory level rule, determines T1 as the motor levels, while the European Multicenter Study about Spinal Cord Injury (EMSCI) ISNCSCI calculator,^7^ which implements the motor follow sensory function rule, determines T5 as right motor level and T4 as left motor level.

**Figure 1** depicts a sample case in which the ML determination is not fully clear. In this case, the sensory levels (SLs) are classified as C5 on the right and C6 on the left side and are within the myotomes with all testable key muscle functions of the upper extremities graded as normal. Additionally, the sensory functions of the adjacent segments T2-T5 (right) and T2-T4 (left) are also graded as normal. Applying the MFSL rule, the right and left motor level is T1.

However, an add-on publication with clarifications^9^ to previous ISNCSCI revisions^8^ suggests to use a generalized MFSF rule for ML determination in not clinically testable segments, where assumptions on segmental motor function are made based on the sensory function of the same segment and body side.^9^ This means that motor function is considered as intact, if both light touch (LT) and pinprick (PP)^10^ sensation are graded as intact. If this generalized MFSF rule is applied to the example case depicted in **Figure 1**, the MLs are classified as T5 (right) and T4 (left). Due to the lack of clear guidance, these rules leave room for interpretation. As an example, the 2 validated computational ISNCSCI calculators (i.e., the Praxis ISNCSCI algorithm calculator^11^ and the European Multicenter Study about Spinal Cord Injury [EMSCI] ISNCSCI calculator^7^,^12^) determine different MLs in cases like the one shown in **Figure 1**.

The aims of this work were to (1) determine the characteristics and frequency of these not fully conclusive cases in a large representative cohort with traumatic SCI using the EMSCI database, and to (2) discuss options for a more conclusive ML definition based on the characteristics of the identified cases.

## Methods

### Identification of conflicting ML determination

Based on the analysis of the case shown in **Figure 1** and similar examples, we identified 3 conditions that, when all are met, result in differing MLs for the MFSL and the MFSF approaches.

1. **Condition 1:** All key muscles of the upper extremity (or upper and lower extremity) on a given side are entirely scored as intact (graded as 5).
2. **Condition 2:** The SL on the side of the body with the intact upper extremity (or upper and lower extremity) is within the segments C4-C8 (or L1-L5) with clinically testable muscle functions.
3. **Condition 3:** A contiguous region with normal light touch (LT) and pinprick (PP) sensation is found, starting one segment caudally to the clinically testable myotomes of the intact extremity (from T2 to L1 or from S2 to S4-5).

If conditions 1 and 2 are present, applying the key muscle rule would result in an ML of T1 (or S1). However, if the MFSF approach is applied, due to condition 3, the ML moves caudally of T1 (or S1)

### Investigated dataset and outcome variables

ISNCSCI datasets from the European Multicenter Study about Spinal Cord Injury (EMSCI) database were used for quantification of the differences in ML determination according to the MFSL and MFSF approaches. As the ML plays a crucial role in the differentiation between AIS B and C/D cases with absent voluntary anal contraction (VAC), we additionally analyzed differences in the AIS between both approaches. Furthermore, the number of intact MLs using the MFSF approach is counted as well as the number of cases with ambiguous MLs at the transition zones.

The representative benchmark dataset was queried in April 2013 from the EMSCI database with the following inclusion criteria additional to the EMSCI inclusion criteria^13^: date of injury between 2006 and 2012, age at injury ≥16 years, traumatic spinal cord injury (tSCI), and acute (less than 30 days after injury) and follow-up (at least 300 days after injury) ISNCSCI assessments available. The EMSCI benchmark dataset consists of 1330 early and late ISNCSCI assessments (NLI: C1-C8 [50.7%], T1-6 [13.7%], T7-12 [23.8%], L1-S5 [11.5%], Intact: 0.8%; AIS: A [40.5%], B [9.8%], C [13.2%], D [35.8%], E [0.8%]) of 665 individuals with traumatic SCI that were documented in 14 European SCI centers.^13^,^14^ If available, associated Spinal Cord Independence Measure (SCIM) III^15^ assessments were also extracted from the EMSCI database.

### Implementation of virtual muscle grades

**Figure 2.**
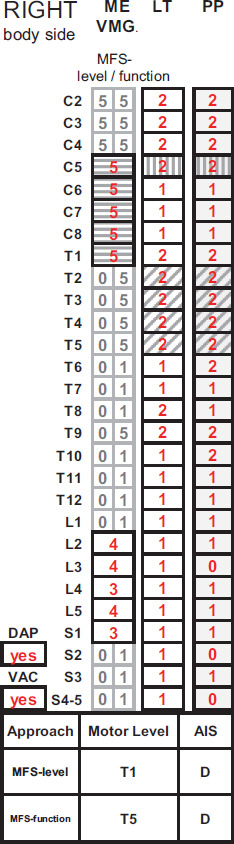
The International Standards for Neurological Classification of Spinal Cord Injury (ISNCSCI) example of Figure 1 (right body side only) with classified motor levels and American Spinal Injury Association Impairment Scale (AIS) grade using the motor follows sensory (MFS)-level approach and the motor follows sensory (MFS)-function approach. Virtual muscle grades (VMG) have been calculated per segment from the ipsilateral light touch (LT) and pinprick (PP) score: VMG is 5, if LT and PP sensations are normal (=2); VMG is 0, if LT and PP sensations are absent (=0); VMG is 1 for all other combinations of LT and PP scores. In the MFSF approach, VMGs are applied to all not clinically testable myotomes: C2-4, T2-L1, and S2-5. In the MFSL approach, VMGs are determined for all not clinically testable myotomes at and rostral to the sensory level, while all VMGs caudal to the sensory level are set to 0. Hatching coding: The sensory level is highlighted with vertical hatching. The extremity with full strength of all key muscle functions is highlighted with horizontal hatching, indicating that the sensory level is within the key muscle myotomes of the extremity. Areas with normal LT and PP sensation below the upper extremity with full strength of all key muscle functions are highlighted with diagonal hatching. It is only when all 3 conditions indicated by the hatching coding are present that the MFSL and MFSF approaches lead to a different motor level. DAP = deep anal pressure; ME = motor examination; VAC = voluntary anal contraction.

The MFSL and the MFSF approaches were implemented in the EMSCI ISNCSCI calculator by using the concept of virtual muscle grades (VMGs).^7^ The VMG concept represents a generalization of the transition zones concept for determination of the ML at C4/C5 (or L1/L2) first specified in 2009 by the International Standards Committee^9^ and defines how a VMG of a not clinically testable myotome (C2-4, T2-L1, and S2-5) is derived from its ipsilateral LT and PP scores:

- A VMG is assumed to be 5 when both LT and PP sensations are graded as normal (e.g., segment T2 in **Figures 1** and **2**).
- The VMG is graded as 0 when both LT and PP sensations are absent (e.g., left segment S2 in **Figure 1**).
- All other LT and PP combinations lead to an impaired VMG of less than 3 (e.g., a VMG of 1), following the guidelines introduced by the ASIA International Standards Committee in the 2009 review^9^ (e.g., the VMG of the segment T6 is graded as 1 in **Figure 2**, with LT in T5 graded as 1 and PP in T6 graded as normal).

In the ML determination process, VMGs were calculated for all not clinically testable myotomes. By this, not only sensory scores but also motor scores are available for classification of all spinal cord segments from C2 to S4-5 (**Figure 2**, ME/VMG). With the introduction of VMGs, the ML can be determined similarly to the less complex process of SL determination, which represents one of the most accurately classifiable ISNCSCI variables.^3^,^16^-^18^

For calculation of the MLs following the MFSL and the MFSF approach, VMGs for clinically not testable myotomes are determined in a different way. For the MFSL approach, VMGs are assumed to be normal for not clinically testable myotomes from C2 to the SL, whereas all VMGs of clinically not testable myotomes caudal to the SL are set to 0. For the MFSF approach, VMGs are calculated for all not clinically testable myotomes according to the definition above (**Figure 2**, MFS-level/function column).

## Results

Motor levels determined by the MFSL or the MFSF approach differ in 34 individuals with 23 of the 1330 right ML (1.73%) and 26 of the 1330 left ML (1.95%) (see eTable). In those 34 individuals, the differences in MLs determined by both approaches have no consequences on AIS grades (4 AIS grade A, 1 AIS grade B, 29 AIS grade D). In the 49 differing MLs, the MLs determined by the MFSL approach are by definition all located at T1 (63.27%) or S1 (36.73%). The MLs determined by the MFSL approach are in all cases at least one segment more rostral to the ones determined by the MFSF approach. The mean difference between the more rostral MFSL-MLs and the MFSF-MLs is 6.76 ± 7.07 segments (MFSL-ML T1: 9.13 ± 7.98 segments; MFSL-ML S1: 2.67 ± 0.77 segments). The mean number of segments between SL and MFSL ML (2.88 ± 1.24) versus SL and MFSF-ML (9.63 ± 7.50), respectively, differs significantly (*P* < .001; Wilcoxon signed-rank test). **Table 1** lists the differences in mean number of segments between the rostral levels and the caudal motor levels determined by the MFSL and the MFSF approaches.

**Table 1.** Distance in number of segments between rostral levels and caudal motor levels (ML) determined by the motor follows sensory level (MFSL) approach and/or the motor follows sensory function (MFSF) approach

Rostral level	Caudal motor level	Distance in number of segments mean ± *SD*
MFSL motor level	MFSF motor level	6.76 ± 7.07
MFSL motor level = T1	MFSF motor level	9.13 ± 7.98
MFSL motor level = S1	MFSF motor level	2.67 ± 0.77
Sensory level	MFSL motor level	2.88 ± 1.24
Sensory level	MFSF motor level	9.63 ± 7.50
Sensory level	MFSL motor level = T1	3.03 ± 1.30
Sensory level	MFSL motor level = S1	2.61 ± 1.04
Sensory level	MFSF motor level with MFSL-ML = T1	12.16 ± 8.43
Sensory level	MFSF motor level with MFSL-ML = S1	5.28 ± 1.36

*Note*: The neurological level of injury always equals the sensory level in these cases.

**Figure 3.**
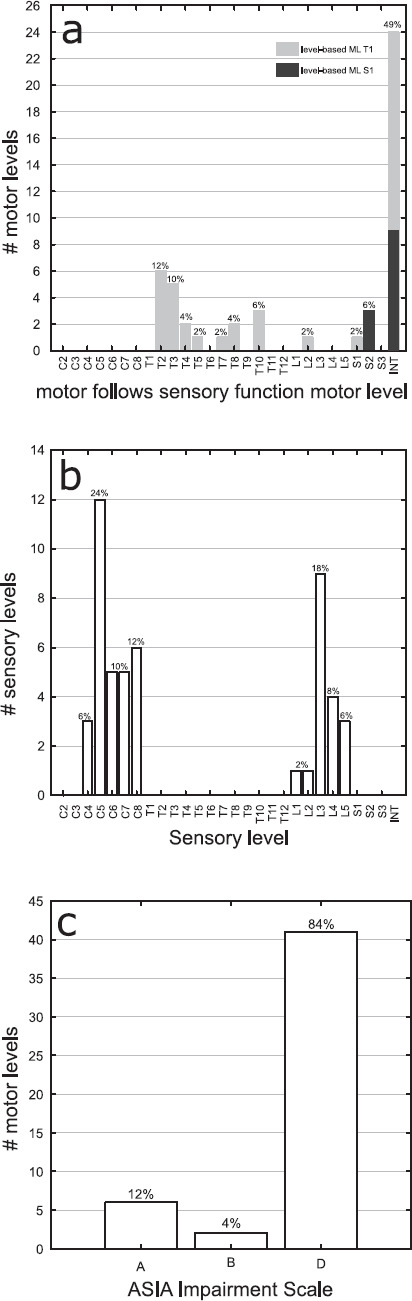
Histogram of all motor levels (MLs) with differences between the motor follows sensory function (MFSF) and motor follows sensory level (MFSL)-based ML determination approaches for the variables. (a) MFSF ML, (b) sensory level, and (c) American Spinal Injury Association (ASIA) Impairment Scale grade. INT = intact.

The majority (24) of the 49 differently classified MLs have an ML determined by the MFSF as intact (49%), with 15 having an ML of T1 and 9 with an ML of S1 according to the MFSL approach (**Figure 3a**).

**Figure 4.**
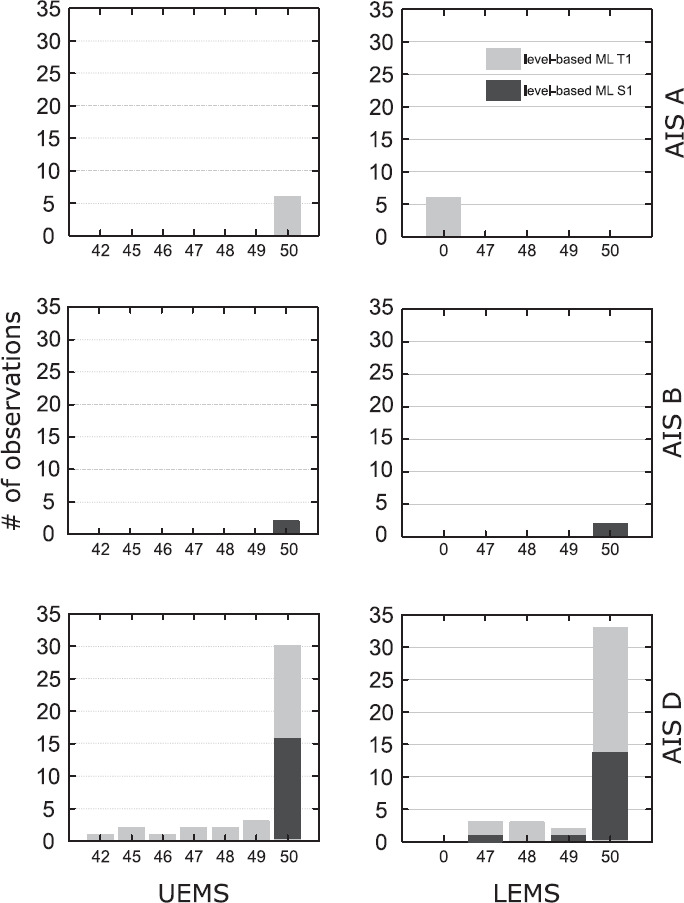
Upper extremity motor scores (UEMS) and lower extremity motor scores (LEMS) grouped by the American Spinal Injury Association Impairment Scale (AIS) grade in rows. The proportions of the corresponding motor follows sensory level–based motor levels (ML) are indicated by a light grey bar for T1 and a dark grey bar for S1.

Two-thirds have a lesion of the cervical spinal cord according to the NLI, which in these cases is determined by the SL (**Figure 3b**), and are classified as AIS D (84%) (**Figure 3c**). In all 6 differently classified MLs from 4 individuals classified as AIS A, full strength in upper extremity key muscles (upper extremity motor score [UEMS] = 50) and no motor function in lower extremity key muscles (lower extremity motor score [LEMS] = 0) are present (**Figure 4**). In one individual with AIS grade B, the MLs on both sides differ for the 2 approaches with full upper (UEMS = 50) and full lower extremities (LEMS = 50) strength. In 29 individuals classified as AIS D, 41 differing MLs were observed, with 10 individuals (for one individual classified as AIS D, 2 exams from different exam stages were included in the analysis) showing ML discrepancies on both sides. In 16 individuals classified as AIS D, no motor deficits according to ISNCSCI (UEMS and LEMS = 50, present VAC) were detectable with a mean total LT score of 103.56 **±** 11.96 (max. 112) and a mean total PP score of 99.69 **±** 15.14 (max. 112). SCIM III assessments are available for 8 of these individuals, with a SCIM total score of 95.67 **±** 3.51 (max. 100) for the 3 individuals with MFSL-ML of T1 and 100 for the 5 individuals with MFSL-ML of S1.

In 15 differing MLs from 12 individuals with an NLI in the cervical segments [15/(2 body sides * 1330 ISNCSCI assessments) = 0.56%], the motor and sensory levels were identified to be in the transition zone C4/C5 with the SL classified as C4 or C5. Two differing MLs were found from 2 individuals with an NLI in the lumbar segments for the lower extremity transition zone L1/L2 with an SL of L1 or L2.

## Discussion

We analyzed the sensorimotor characteristics of cases where difficulties arise in the ML classification according to the ISNCSCI. This is the case, if (1) the SL is within myotomes of an upper (i.e., C4-T1) or a lower (i.e., L1-S1 with upper extremity myotomes being intact) extremity graded entirely as intact or and (2) a contiguous region with normal sensation starts at T2 (or S2). Although these cases exist, they occur very rarely. They were found in only less than 4% of all investigated MLs of ISNCSCI records from the representative EMSCI cohort, predominantly in people with very incomplete lesions. There are multiple conditions that might lead to such an impairment pattern: An obvious explanation would be an isolated impairment of afferent spinal tracts with preservation of efferent pathways. Second, isolated sensory impairments with normal sensation in the surrounding segments may be exclusively caused by an above-level non-SCI condition, such as a sensory polyneuropathy. If there are any doubts about the involvement of the peripheral nervous system in such a non-SCI condition, additional tests such as neurophysiological measurements of nerve conduction velocities should be performed. Once it has been confirmed that the non-SCI condition is solely responsible for the isolated sensory impairment, the recently introduced non-SCI taxonomy^19^ mandates that the not-normal sensory scores should be tagged with an asterisk. The non-SCI condition (e.g., peripheral nerve injury) along with an explicit note that the abnormal scores are handled as normal for classification purposes must be documented in the comments box. Third, the pattern can be caused by multiple spinal cord lesions. In such cases it has been agreed to not provide an AIS grade, however sensory and motor levels of the most rostral SCI should be given.^20^ Fourth, random errors during examination and paper documentation, or during transcription of scores from paper sheets into the electronic database, could be a cause of this rare, unusual neurological impairment pattern. However, we consider this to be a minor contributor to the issue. Fifth, for a nonexperienced examiner, it might be challenging to distinguishing between normal and altered sensory function, particularly in very incomplete injuries. However, all of the ISNCSCI examiners in the EMSCI network were trained.^3^

Our results show that most (84%) of these rare cases are classified as AIS D with no measurable deficits in clinically testable key muscle functions. This leads to an intact ML grading when using the MFSF approach (eTable). Our analysis of the functional independence, as assessed by the SCIM III in some of these cases with no detectable deficit in the ISNCSCI motor exam, indicates that completely intact motor functions might be present in cases with NLI in the lumbar segments where the NLI equals the SL. For cases with a sensory level (equals NLI) in the cervical region, the total SCIM III scores did not reach their maximum, which might indicate the presence of not only of sensory deficits but also of motor functions not tested as part of the ISNCSCI exam. However, due to the very small size of this subgroup (*n* = 3), no definite conclusions can be made on whether MLs determined by the MFSL or the MFSF approach better reflect the clinical presentation of these cases. A more thorough investigation of patients presenting with such an unusual sensory impairment pattern is needed. This includes testing of non-ISNCSCI key muscles, checking for the presence of non-SCI conditions such as sensory polyneuropathy, and verifying potential documentation errors.

Nevertheless, the current motor level definition is not fully conclusive and needs refinement to provide examiners clear guidance in the classification and evaluation of the cases presented in this work. The most conservative option for ML determination in not clinically testable segments is the motor follows sensory level approach. This approach generally results in more rostral motor level with less distance to the SL. However, the current motor follows sensory level definition is not fully compliant with the recommended procedure of ML determination at the transition zones C4/C5 and L1/L2, respectively.^9^ Therefore, a refined ML definition based on components from both the MFS function as well as the MFSL approaches might be advisable, which assumes (1) that motor function in not clinically testable myotomes rostral to and at the sensory level to be normal (following the concept of the MFSF approach) and (2) that not normal motor function is present caudal to the sensory level (following the MFSL approach).

During the implementation of the MFSL and MFSF approaches into the EMSCI ISNCSCI calculator, we refined the ML determination approach at transition segments into the more general concept of deriving VMGs from sensory scores in not clinically testable myotomes. This assumes that for not clinically testable myotomes (a) motor function above and at the sensory level is normal and (b) motor function below the sensory level is not normal (motor score <3; e.g., 1). We found this concept extremely helpful not only for use in computer algorithms but also for manual classification. We would therefore recommend teaching the basics of this concept in training courses.

## Limitations

This work has some limitations that are mostly related to the small number of cases with the identified conflicting conditions, particularly the low availability of SCIM assessments. The ISNCSCI has the inherent limitation that motor functions are only clinically testable in in 10 myotomes, even though the range of motor levels is expected to cover all spinal segments. To address this, the motor level follows the sensory level concept was introduced for cases where the motor level is expected to be in a region with no clinically testable motor functions. Although the validity of motor levels based on assumptions from sensory functions outside the 10 testable myotomes remains conceptually debatable, it provides clinically meaningful information about the segmental level below motor impairments might be present. However, a discussion about the conceptional limitations of the ISNCSCI classification goes well beyond the scope of this article.

## Conclusion

With this work, we identified and characterized rare cases of an unusual neurological impairment pattern with normal key muscle functions in an upper (or both upper and lower) extremity but not normal sensation at a key muscle segment, however, with a contiguous region with normal sensation starting at T2 (or S2). In these cases, the motor level definition in the current ISNCSCI revision is not fully conclusive.

An obvious explanation for this pattern would be an isolated impairment of afferent spinal tracts with preservation of efferent pathways. However, non-SCI conditions such as sensory polyneuropathy might exclusively cause such isolated sensory impairments with normal sensation in the surrounding segments. Additional assessments are recommended to confirm that the non-SCI condition is solely responsible for the isolated sensory impairment.

Nevertheless, a refinement of the current ML definition is necessary to provide better guidance to examiners in the complex task of determining the ML in the identified cases is necessary.

## Supplementary Material


