# Supplementary material for: Clarifications of the Motor Level Definition in the International Standards for Neurological Classification of Spinal Cord Injury in Not Clinically Testable Myotomes
Source: Top Spinal Cord Inj Rehabil. 2025 Aug 22;31(3):37–47. doi: 10.46292/sci24-00094 (PMC12376153; doi:10.46292/sci24-00094)
Supplement: Supplementary file 1 [file i1945-5763-31-3-37_s01.pdf]

eTable. Characteristics of cases with different motor levels using the motor follows sensory function and the motor follows sensory level rules

Number	ID	Age range, years	Exam stage	Body side	Motor follows sensory function–based motor level	Motor follows sensory level–based motor level	Sensory level	AIS	UEMS	LEMS	PP	LT	SCIM III
1	xxx098	71-75	chronic	right	T7	T1	C5	D	50	50	89	112	
2	xxx108	41-45	chronic	left	T3	T1	C8	A	50	0	41	40	
3	xxx108	41-45	chronic	right	T3	T1	C8	A	50	0	41	40	
4	xxx141	21-25	chronic	right	INT	S1	L3	D	50	50	110	107	100
5	xxx141	21-25	chronic	left	INT	S1	L4	D	50	50	110	107	100
6	xxx158	21-25	very acute	left	T2	T1	C4	D	49	50	90	86	
7	xxx184	16-20	chronic	right	INT	S1	L4	D	50	50	110	110	100
8	xxx257	81-85	acute I	left	INT	S1	L4	D	50	50	108	101	
9	xxx500	31-35	acute I	left	S2	S1	L3	B	50	50	102	108	
10	xxx500	31-35	acute I	right	S2	S1	L3	B	50	50	102	108	
11	xxx084	26-30	chronic	right	T2	T1	C8	A	50	0	36	37	
12	xxx090	21-25	chronic	left	S2	S1	L5	D	50	47	95	99	
13	xxx093	16-20	chronic	left	INT	S1	L5	D	50	50	110	110	100
14	xxx093	16-20	chronic	right	INT	S1	L5	D	50	50	110	110	100
15	xxx278	46-50	very acute	left	T8	T1	C5	A	50	0	56	57	
16	xxx278	46-50	very acute	right	T8	T1	C5	A	50	0	56	57	
17	xxx298	46-50	chronic	right	T3	T1	C4	D	42	47	77	94	
18	xxx419	71-75	chronic	left	INT	S1	L3	D	50	50	109	112	
19	xxx436	71-75	chronic	right	T5	T1	C4	D	50	50	57	92	96
20	xxx027	41-45	chronic	left	T10	T1	C7	D	50	50	90	90	
21	xxx027	41-45	chronic	right	T10	T1	C7	D	50	50	90	90	

(*continues*)

This supplementary digital material accompanies
Top Spinal Cord Inj Rehabil 2025;31(3):37-47
© 2025 American Spinal Injury Association
www.asia-spinalinjury.org
doi: 10.46292/sci24-00094

eTable. Characteristics of cases with different motor levels using the motor follows sensory function and the motor follows sensory level rules *(cont.)*

Number	ID	Age range, years	Exam stage	Body side	Motor follows sensory function–based motor level	Motor follows sensory level–based motor level	Sensory level	AIS	UEMS	LEMS	PP	LT	SCIM III
22	xxx064	56-60	chronic	left	T10	T1	C6	D	48	49	78	95	
23	xxx105	46-50	chronic	right	INT	T1	C5	D	50	50	103	109	99
24	xxx105	46-50	chronic	left	INT	T1	C5	D	50	50	103	109	99
25	xxx147	25-30	additional	right	S1	T1	C5	D	50	50	110	105	
26	xxx147	25-30	additional	left	INT	S1	L3	D	50	50	110	105	
27	xxx158	16-20	chronic	left	INT	T1	C5	D	50	50	112	108	
28	xxx158	16-20	chronic	right	INT	T1	C5	D	50	50	112	108	
29	xxx186	51-55	chronic	right	T2	T1	C5	D	47	48	105	65	
30	xxx210	56-60	chronic	right	T2	T1	C5	D	50	50	76	66	
31	xxx253	31-35	acute I	right	T4	T1	C8	A	50	0	42	43	
32	xxx261	36-40	chronic	right	INT	T1	C5	D	49	50	105	106	
33	xxx298	36-40	chronic	right	T4	T1	C7	D	46	47	55	104	
34	xxx341	41-46	chronic	left	INT	S1	L1	D	50	50	101	108	100
35	xxx341	41-46	chronic	right	INT	S1	L4	D	50	50	101	108	100
36	xxx377	71-76	chronic	left	INT	T1	C6	D	50	50	107	110	92
37	xxx377	71-76	chronic	right	INT	T1	C6	D	50	50	107	110	92
38	xxx424	66-70	chronic	left	T3	T1	C5	D	45	48	71	106	
39	xxx020	16-20	chronic	right	INT	T1	C6	D	47	50	108	111	
40	xxx102	16-20	acute I	left	INT	S1	L3	D	50	50	106	106	
41	xxx102	16-20	acute I	right	INT	S1	L3	D	50	50	106	106	
42	xxx102	16-20	chronic	left	INT	S1	L3	D	50	50	105	106	100
43	xxx102	16-20	chronic	right	INT	S1	L3	D	50	50	105	106	100
44	xxx118	46-50	chronic	right	T3	T1	C7	D	49	50	64	98	

(*continues*)

eTable. Characteristics of cases with different motor levels using the motor follows sensory function and the motor follows sensory level rules *(cont.)*

Number	ID	Age range, years	Exam stage	Body side	Motor follows sensory function–based motor level	Motor follows sensory level–based motor level	Sensory level	AIS	UEMS	LEMS	PP	LT	SCIM III
45	xxx039	36-40	chronic	right	INT	T1	C7	D	50	50	97	111	
46	xxx039	36-40	chronic	left	T2	T1	C8	D	50	50	97	111	
47	xxx106	36-40	chronic	left	INT	S1	L2	D	50	49	107	104	
48	xxx109	21-25	chronic	left	T2	T1	C8	D	48	50	109	112	
49	xxx014	21-25	chronic	left	L2	T1	C6	D	45	48	107	112	

Note: AIS = ASIA Impairment Scale; ASIA = American Spinal Injury Association; ID = individual; INT = intact; LEMS = lower extremity motor score; LT = light touch, total score; PP = pinprick, total score; SCIM = Spinal Cord Independence Measure; UEMS = upper extremity motor score.
